# Clinical Neuropathology teaching case 3-2015: female or male brain? Anti-ubiquitin visualizes Barr bodies in hippocampal granule cells which allows the determination of gender in human brains 

**DOI:** 10.5414/NP300877

**Published:** 2015-04-24

**Authors:** Ellen Gelpi

**Affiliations:** Neurological Tissue Bank of the Biobanc-Hospital Clinic-Institut d’Investigacions Biomediques August Pi i Sunyer (IDIBAPS), Barcelona, Spain

**Keywords:** Barr bodies, ubiquitin, hippocampus

## Abstract

No Abstract available.

Anti-ubiquitin immunohistochemistry is widely used for routine neuropathological work-up of neurodegenerative diseases. The hippocampus is one of the important screening areas of pathology in the human brain. In addition to the detection of dystrophic neurites of amyloid plaques, neurofibrillary tangles, Pick bodies, argyrophilic grains, neuronal cytoplasmic inclusions in fronto-temporal lobar degeneration, glial pathology in progressive supranuclear palsy, corticobasal degeneration, and multiple system atrophy, the hippocampal area has proven very useful for the detection of potential C9orf72 hexanucleotide repeat expansion mutation carriers [1]. 

In addition to the labelling of pathological structures, anti-ubiquitin immunohistochemistry may also visualize physiological structures that undergo ubiquitination, such as Barr bodies. Barr bodies represent the inactive X chromosome in somatic cells of females [2], therefore they are usually not observed in males except for individuals who have an extra X chromosome as it is the case in Klinefelter syndrome (XXY). Barr bodies are seen at the edge of the nucleus as a small spherical dark condensation attached to the nuclear membrane [2, 3]. They can be observed by means of conventional histochemical stains that visualize the cell nucleus, such as hematoxylin-eosin, Cresyl violet or Papanicolaou stains, or with the help of fluorescent dies [3]. Mechanisms of compactation of Barr bodies include histone H3 methylation and histone H2A ubiquitination [4, 5, 6]. 

Using anti-ubiquitin immunohistochemistry, Barr bodies can be nicely visualized in the human brain in hippocampal granule cells as illustrated in Figure 1, which allows for the determination of gender in human brains. 

## Conflict of interest 

The author reports no conflict of interest. 

**Figure 1.**
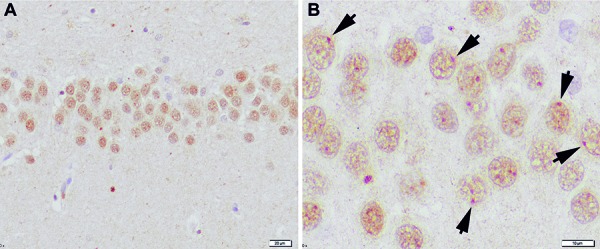
Anti-ubiquitin immunohistochemistry on human hippocampus. A: low magnification of granule cells of dentate gyrus showing diffuse nuclear labelling. B: higher magnification depicts small ubiquitin-positive spherical condensations at the edge of the nuclei (arrows). Nucleoli are not stained.
